# Influence of different alcohol intake frequencies on alveolar bone loss in adult rats: A sem study

**DOI:** 10.4317/jced.54647

**Published:** 2018-09-01

**Authors:** Daniela-Martins de Souza, Vinicius-Anéas Rodrigues, Alan-de Aquino Silva, Vitor-Sulz Gonsalves, Kauê-Alberto Pereira, Renato-Sussumu Nishioka, Claudemir de Carvalho

**Affiliations:** 1 DDs, MSc, PhD, Professor, College of Pindamonhangaba, Christian Life University Foundation-FUNVIC. Addres: Rua Marechal Deodoro da Fonseca, 316 – Centro, Pindamonhangaba, São Paulo, Brazil; 2 DDs, College of Pindamonhangaba, Christian Life University Foundation-FUNVIC. Addres: Rua Marechal Deodoro da Fonseca, 316 – Centro, Pindamonhangaba, São Paulo, Brazil; 3 DDs, MSc, PhD, Adjunct Professor, Department of Dental Materials and Proshodontics, São Paulo State University (Unesp), Institute of Science and Technology, Institute of Science and Technology, São José dos Campos / SP, Brazil. Address: Av Engenheiro Francisco José Longo, 777, Jardim São Dimas, São José dos Campos, São Paulo, Brazil; 4 MSc, PhD, Professor, College of Pindamonhangaba, Christian Life University Foundation-FUNVIC. Addres: Rua Marechal Deodoro da Fonseca, 316 – Centro, Pindamonhangaba, São Paulo, Brazil

## Abstract

**Background:**

Alcohol intake is associated with oral diseases and bone changes including alveolar bone loss in humans and in experimental animals. The main aim of the present study is to assess the effect of long-term alcohol intake, at different frequencies, on periodontal bone loss (PBL) in adult rats.

**Material and Methods:**

Thirty-six (36) rats were divided into 3 groups: Control (daily water intake, n=12), daily alcohol intake (20% ethanol, n=12), and social alcohol intake (20% ethanol twice a week, n=12). The rats were sacrificed after 90 days and their right maxillae were removed. Initially, a random portion from each group was analyzed through SEM (scanning electron microscope) to assess surface topography. Next, all pieces were dissected and stained with methylene blue 1% and photographed in stereomicroscope at 10x magnification. The PBL was assessed by measuring the distance between cement-enamel junction and alveolar bone crest.

**Results:**

Results showed higher (*p*=0.0368) alcohol solution amount in the daily intake group than in the twice week intake one. The SEM showed qualitatively flat bone surface in the control group, the social intake group presented surface with few minor hollows, and the daily intake group evidenced increased number and diameter of wells. The comparison between groups showed higher bone loss (*p*<0.05) in both frequencies than in the control, but the bone loss was lower (*p*<0.05) in the social alcohol intake group than in the daily intake one.

**Conclusions:**

Alcohol intake may cause alveolar bone loss in periodontitis-free rats depending on the frequency.

** Key words:**Alcohol intake, alveolar bone loss, alcohol-induced periodontitis, alcoholic rats.

## Introduction

Many diseases derive from alcohol consumption, it also affects the well-being and health of people living close to alcohol users. The amount of consumed alcohol is particularly important when the association between alcohol and chronic diseases is taken into account. Constant alcohol consumption may be associated with increased risk of developing diseases due to the close relation between alcohol intake and the incidence of certain diseases (1). Alcohol consumption is the causal component of more than 200 diseases and harmful conditions, mostly of alcohol dependence, liver cirrhosis and cancers (2).

Acute and chronic excessive alcohol consumption has serious effect on oral health, besides its association with inappropriate oral hygiene and diminished salivary flow. It also increases the risk of oral cancer, dental caries, teeth loss and periodontitis (3).

Chronic alcoholic patients show increased risk of developing severe infection possibly caused by altered immune response. Moreover, the toxic effect alcohol has on the liver leads to negative impacts on the clotting mechanism (4).

Adult human maxilla and mandible can be subdivided by an alveolar process that involves housing the roots of erupted teeth and the basal body. The alveolar processes consist of the thin alveolar bone proper, which forms an alveolar wall on the tooth socket, inner and outer cortical plates and on the spongy bone between the alveolar bone proper and the cortical plates (5).

Mineralized tissue resorption by the alveolar bone requires the recruitment of specialized cells called osteoclasts. These cells are produced by the monocyte/macrophage strain of hematopoietic cells deriving from the bone marrow. Alveolar bone formation seems to be linked to bone resorption. It happens in order to maintain bone mass, and involves stromal stem cell proliferation and differentiation along the osteogenic pathway; such proliferation leads to osteoblast formation. The cellular differentiation process is controlled by a number of events involving the combination among the genetic programming and gene regulation performed by different hormones, cytokines and growth factors (6).

Alcohol intake can directly affect bone metabolism, since it causes tissue-formation suppression due to its toxic effect on the osteoblastic activity and proliferation (4). Moreover, alcohol does not have direct effect on the bone, but it can worsen bone loss by modulating the hormones that regulate bone metabolism (7).

Rats are often used in models of experimental periodontitis because periodontal anatomy in the molar region shares some similarities with that of humans (8).Therefore, rat models have been used to periodontal pathogenesis experiments (8) and methodological study of periodontium (9,10).

They have been used in investigations about the influence of potential risk indicators/factors of diabetes (11), simultaneous nicotine and alcohol use (12), alcohol consumption (13,14) and estrogen deficiency (15,16), on periodontium alterations, besides their use to assess changes in the mandibular calcium and phosphorus concentrations in rats (17). The SEM (scanning electron microscope) method has been more often used to assess dental and periodontal structures (17,18).

Most studies testing the hypothesis that alcohol intake may increase alveolar bone loss were conducted in rats presenting induced periodontitis; therefore, the aim of the current study was to assess the effect of long-term daily and social alcohol intake on bone loss in adult rats without ligature-induced periodontitis.

## Material and Methods

-Materials

The total of 36 male Wistar rats were provided by the animal house of Pindamonhangaba School. They were acclimatized for 10 days before the experiment and housed in controlled-temperature (24 ± 2ºC) room under a 12-hour light/dark cycle (7pm to 7am).The animals were fed with proper chow and filtered water ad libitum. The experimental model was carried out according to international ethical principles for animal studies in laboratory. The study was approved by the Institutional Animal Research Committee (Protocol CEEA 025/2013).

-Methods

Experimental groups 

The rats were randomly distributed into three groups: Control (daily water supply, n=12), daily alcohol intake (20% alcohol, n=12), and social alcohol intake (20% alcohol twice a week, n=12). The liquid diet consisted of 20% alcohol solution provided in a daily basis or twice a week for 90 days.

Sample size calculations

Sample size estimates were calculated based on data of a previous study (13). A difference in 0.2mm in alveolar bone loss was expected as significant. Considering alpha and a beta errors of .05 and .20 respectively, a minimum number of nine rats per group was considered necessary (19).

Analysis

After the animals were subjected to euthanasia, their maxillae were immersed in 10% formalin solution for 48 hours, washed in running water and air-dried.

Initially, a randomized portion of each group was analyzed in five sites through SEM (scanning electron microscope, Inspect S50, FEI, Czech Republic) in order to assess surface topography.

The alveolar bone was prepared for SEM analysis by dehydrating three specimens in alcohol at increasing concentrations (60%, 70%, 80%, 90% and 100%). The dehydration process consisted in immersing the specimens in each of the aforementioned solution concentrations for 2 hours. Subsequently, the specimens were kept at the final concentration (100%) for 24 hours until the end of the process. Next, they were air-jet dried and stored in a desiccator containing silica gel until they were processed to perform the analysis (17). This procedure decreased noise during the SEM analysis applied to the specimens. A thin gold layer was deposited on the surface by using a vacuum depositor because of the specimen’s low electric conductivity.

**Figure 1:**
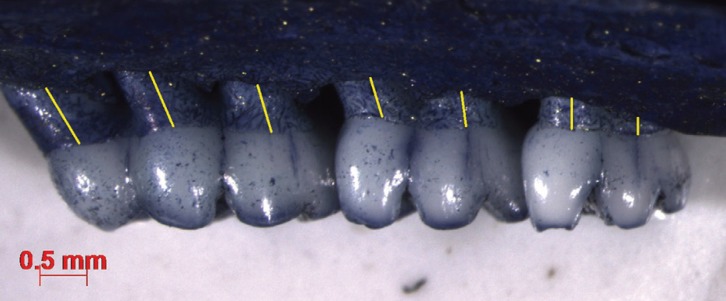
Photograph of maxilla lingual aspect taken in stereomicroscope (X10). The lines indicate the seven distances between cement-enamel junction (CEJ) alveolar bone crest measured in the teeth.

All right maxillae were stained with methylene blue solution 1% and photographed and measured in stereomicroscope (Stereo Discovery V20, Zeiss, Germany) at 10x magnification (Carl Zeiss Axiovision Rel.4.6.Germany). Periodontal bone loss (PBL) was morphometrically assessed by measuring the distance between cement-enamel junction (CEJ) and alveolar bone crest in seven (7) different palatal sites of each maxilla (Fig. 1).

All measurements were taken along the long axis of the roots and from the palatal side. Bone level was set as the mean of these seven distances per tooth. The mean of these measurements was used as alveolar bone loss measurement in each group. All measurements were blindly conducted in the group the rat belonged to.

The teeth should not interproximally overlap each other and the buccal cusp tip of each molar should be superimposed to the corresponding lingual cusp tip in order to achieve sufficient molar alignment reproducibility in the image.

Reproducibility

Double measurements were randomly taken in twelve specimens by a single examiner in one-week intervals. The intra-class correlation coefficient (ICC) between measurements revealed a very high correlation (ICC = 0.96).

Statistical analysis

The liquid diet was analyzed as the mean and standard deviation of each alcohol consumption group throughout the experimental period. The independent t-test (*p*<0.05) sample was used to compare the twice-a-week intake group to the daily alcohol intake one.

Numerical data were explored for normality by checking the data distribution and using Kolmogorov-Smirnov test. Normal distribution was detected (*P*-Value >0.150) and showed parametric distribution. The data were represented as mean, standard deviation and 95% Confidence interval value. One-way ANOVA test was used to compare between the three groups. Tukey’s post-hoc test was used for pair-wise comparisons when ANOVA test was significant. The significance level was set at (*p*< 0.05) and showed significant PBL differences between the control and tests groups.

## Results

-Diet Analysis

The daily alcohol intake group consumed 58.08±16.16 ml/d of alcohol solution, on average. The twice-a-week alcohol intake group consumed 47.34±13.03 ml/d of alcohol solution, on average. Results showed higher (*p*=0.0368) consumption in the daily alcohol intake group than in the social alcohol intake one.

Morphometric Analysis

**Figure 2:**
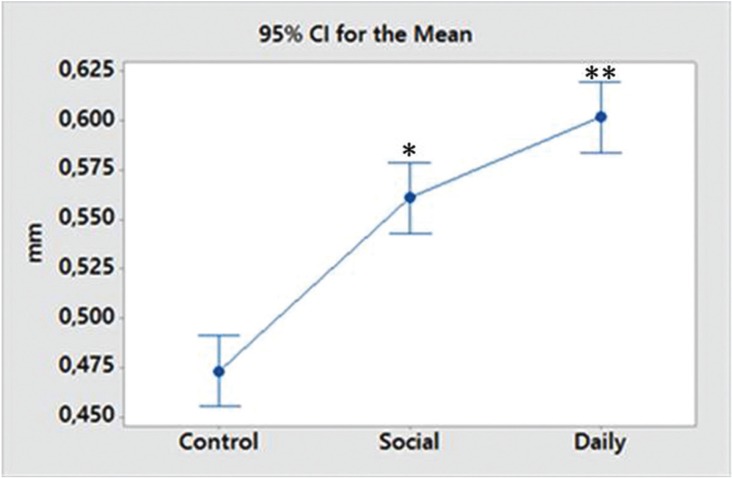
Mean and standard derivation of the periodontal bone loss (mm) in each group. *Statistically significant (ANOVA and Tukey test; *p*<0.05).

The results showed higher (*p*<0.05) bone loss in the social (0.560 ± 0.037 mm) and daily alcohol intake (0.601 ± 0.024 mm) groups when they were compared to the control (0.473 ± 0.027 mm). Bone loss was lower (*p*<0.05) in the social alcohol intake group than in daily intake one (Fig. 2).

-SEM Analysis

**Figure 3:**
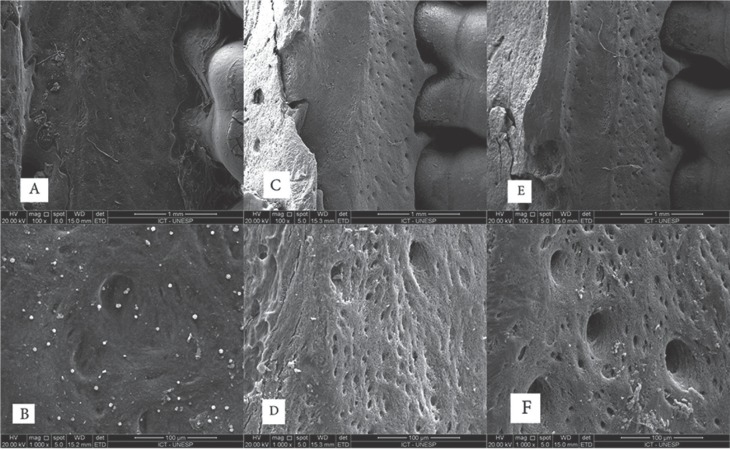
Images taken in scanning electron microscope and used to assess the surface topography showing qualitatively flat bone surface at 100X and 1000X magnification. Reduced number of porosity and diameter in the Control group, figures: a) 100x and b) 1000x. Moderate number of porosity and diameter in the social alcohol intake group, figures: c) 100x and d) 1000x. Larger number of porosity and diameter in the daily alcohol intake group, figures: e) 100x and f) 1000x.

**Figure 4:**
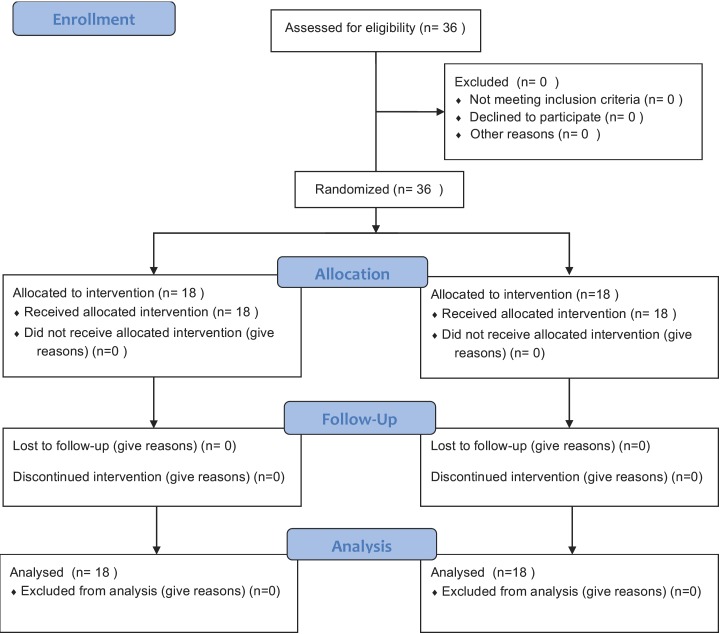
CONSORT 2010 Flow Diagram.

The SEM showed qualitatively flat bone surface: reduced porosity and diameter in the Control group (Fig. 3A,B), moderate porosity and diameter in the social alcohol intake group (Fig. 3C, D) and higher porosity and diameter in the daily alcohol intake group (Fig. 3E, F, 4).

## Discussion

Alcohol is a psychoactive substance presenting dependence-producing properties that have been widely used in many cultures for centuries. The harmful alcohol consumption leads to a large number of diseases, as well as worsens social and economic burdens for society (2).

Rat models have been used in investigations about the influence of potential risk indicators/factors of alveolar bone loss such as height (11-14,16,19) and changed mandibular Ca and P concentrations (17). Alcohol consumption during pregnancy implies many complications, among them, buccal alterations related to bone tissue changes in newborns. Alcohol exposure before and throughout the gestational period reduces mandibular calcium and phosphorus concentrations in newborn rats (17).

The present study was conducted to assess the effect of long-term daily and social alcohol intake (90 days) on alveolar bone loss in male rats. Results evidenced that both frequencies worsened periodontal bone damages, but the social alcohol intake (twice a week) group presented less bone loss than the daily intake group.

There is a dose-response association between alcohol intake volume and many diseases and injuries (2). The dose-dependent relation linked to alveolar bone loss appears to be consistent with its biological plausibility. With regard to the present study, the frequency and volume of consumed alcohol were directly associated to each other, i.e., less amount of consumed alcohol corresponded to lower frequency, and the same applies to then opposite.

Previous studies conducted with rats have shown that long-term alcohol consumption - 4 month (120 days) (20) or 100 days (21); mid-term consumption - 63 days (9 weeks) (19),55 days (22) or 56 days (8 weeks) (13,23), and short-term consumption -28 days or 4 weeks (14,24) was associated with increased alveolar bone loss severity on the absence of periodontitis (14,22) or of induced periodontitis (13,15,20,21,23,24) .

However, as far as it is known, the present study in pioneer in addressing the association among alveolar bone loss, different alcohol intake frequencies (90 days) and periodontitis absence. It is worth highlight that reduced alcohol intake frequency or amount (social intake) may result in bone damage, however the damages are milder when the intake frequency is lower. With regard to the current study, the daily alcohol intake was associated with higher amounts of ingested alcohol, in comparison to the social and twice-a-week intake groups. The amount of ingested alcohol was directly proportional to consumption frequency.

Moderate, but consistent, dose-dependent relation between alcohol consumption and increased periodontal disease severity was recorded by an important cross-sectional study (25), involving 13.198 individuals. Another study (24) evidenced that alcohol consumption effects may modulate periodontal bone loss in ligature-induced periodontitis in female rats directly depending on the dose, i.e., the higher the dose, higher the bone loss and vice-versa.

Irie *et al.* (14) showed that the distance between cement-enamel junction and alveolar bone crest was greater in the alcohol intake group in absence of induced periodontitis than in the control group. Bannach *et al.* (22) showed that the distance between cement-enamel junction and alveolar bone crest of the palatal site in the first mandibular molar was greater in the alcohol intake group than in the control group in absence of periodontitis.

Some studies (13,15,21,23,24) showed that alcohol intake itself may not cause bone loss in rats’ periodontium. On the other hand, other studies (14,22) recorded that chronic alcohol consumption itself may cause bone loss in rats, and this assumption is corroborated by results in the present study.

Studies about the possible mechanisms involved in bone metabolism, as well as longitudinal studies, are required to confirm the role of alcohol consumption as alveolar bone loss aggravator.

In conclusion, long-term daily and social alcohol intake may cause alveolar bone loss in periodontitis-free rats depending on the frequency.
